# Recent advances in understanding West Nile virus host immunity and viral pathogenesis

**DOI:** 10.12688/f1000research.13362.1

**Published:** 2018-03-19

**Authors:** Huanle Luo, Tian Wang

**Affiliations:** 1 Department of Microbiology & Immunology, University of Texas Medical Branch, Galveston, USA; 2 Department of Pathology, University of Texas Medical Branch, Galveston, USA; 3 Institute for Human Infections & Immunity, University of Texas Medical Branch, Galveston, USA

**Keywords:** West Nile Virus, Pathogenesis, Animal models, Host Immunity

## Abstract

West Nile virus (WNV), a mosquito-borne flavivirus, has been a significant public health concern in the United States for nearly two decades. The virus has been linked to acute viral encephalitis, neurological sequelae, and chronic kidney diseases. Neither antiviral drugs nor vaccines are currently available for humans. *In vitro* cell culture and experimental animal models have been used to study WNV infection in humans. In this review, we will focus on recent findings and provide new insights into WNV host immunity and viral pathogenesis.

## Introduction

West Nile virus (WNV), a mosquito-borne, single-stranded, positive-sense flavivirus, has been a significant public health concern in the United States for nearly two decades. It was originally isolated in Uganda in 1937 and later caused epidemics in Africa, Europe, the Middle East, and parts of Asia. The virus was introduced to the United States in 1999, and since then it has caused more than 46,000 confirmed human cases and about 2,000 deaths ^1, 2^. While the majority of human infections are asymptomatic, about 20% of infected individuals become symptomatic and develop flu-like symptoms such as rash, headache, myalgia, and gastrointestinal discomfort. Less than 1% of all infected people develop severe neurological disease, including encephalitis, meningitis, acute flaccid paralysis, and death. Up to 50% of WNV convalescent patients develop persistent neurological sequelae or chronic kidney diseases ^2– 5^. Currently, neither treatments nor approved vaccines are available for use in humans to protect against WNV infection. Both *in vitro* cell culture and experimental animal models have been used to study WNV infection in humans. In this review, we will mainly focus on the findings made within the last five years and provide new insights into WNV host immunity and viral pathogenesis.

## Host immunity

### Innate immunity

WNV activates the signaling pathways of several pathogen recognition receptors (PRRs), including Toll-like receptors (TLRs) 3 and 7, RIG-I-like receptors (RLRs), and NOD-like receptors containing pyrin domain (NLRPs), in order to boost innate immunity and culminate in the synthesis of antiviral cytokines, including type I interferons (IFNs), proinflammatory cytokines and chemokines ^6– 9^. The cytosolic DNA sensor cyclic GMP–AMP synthase (cGAS) is also pivotal in protecting the host from WNV infection, though the underlying mechanism has not been defined ^10^. TLR8, the natural ligand for which remains unknown, associates with suppressor of cytokine signaling 1 (SOCS-1) and inhibits the TLR7-mediated antiviral immune response to facilitate WNV infection in mice ^11^.

IFN responses contribute to host defense mainly in two ways. First, IFNs and IFN-stimulating genes (ISGs), including *Ifit2*, *Ifi27l2a*, and *Ifitm3*, participate in the control of WNV infection, prevent the virus from invading the central nervous system (CNS), and restrict its spread in the brain ^12– 16^. Second, both type I and type III IFNs (IFN-λ) are implicated in promoting blood–brain barrier (BBB) integrity, which may prevent WNV entry to the CNS. Studies in murine models suggest that type I IFNs are directly involved in the permeability of the endothelium and the formation of tight junctions through balanced activation of Rac1 and RhoA—small guanosine triphosphatases (GTPases)—interactions and indirect suppression of the compromise effects of proinflammatory cytokines ^17^. More recently, Daniels *et al*. reported that type I IFNR signaling in astrocytes regulates BBB permeability and protects the cerebellum from WNV infection and immunopathology ^18^. Furthermore, the activation of TAM receptor Mertk synergizes with IFN-β to tighten cell junctions and prevent WNV transit across brain microvascular endothelial (BMVE) cells. As a consequence, mice deficient of Mertk were highly vulnerable to a neuroinvasive WNV strain infection ^19^. IFN-λ signaling also modulates tight junction protein localization in a signal transducer and activator of transcription 1 (STAT1)-independent manner in mouse BMVE cells, which leads to a rise in transendothelial electrical resistance and a fall in the movement of virus across the BBB during WNV infection ^20^. Multiple host and viral factors have been reported to be involved in regulating IFN signaling during WNV infection. For example, IRF-3, -5, and -7 are the key transcription factors responsible for mediating type I IFN and ISG responses downstream of RLR signaling in WNV-infected myeloid dendritic cells (DCs) ^21^. Another transcription factor, ELF4, is recruited by STING following WNV infection and interacts with the mitochondrial antiviral-signaling protein (MAVS)–TBK1 complex, which is critical for further induction of type I IFN responses ^22^. Both PI3K/Akt and microRNA miR-34a inhibit WNV infection by positively regulating type I IFN signaling ^23, 24^. Several factors also contribute to the negative regulation of IFN responses. Among them, the activating signal cointegrator complex 3 (ASCC3) protein functions to suppress ISG expression in an IRF3- and IRF7-dependent manner ^25^. UBXN1, a UBX-domain-containing protein family member, can bind to MAVS, the central adaptor protein to RLR signaling, and disrupt IFN-mediated antiviral immune responses ^26^. The nonstructural protein NS1 of WNV is secreted upon infection and associates with and represses TLR3-induced IFN responses in both human and mouse cells ^27^. Among the proinflammatory cytokines induced during WNV infection, interleukin (IL)-1β was shown to synergize with type I IFN and suppress WNV replication in mouse cortical neurons ^9^. Systems immunology studies in cohorts of human subjects with a history of WNV infection also reveal IL-1β induction as a predictive signature of susceptibility to WNV infection ^28^. Several chemokines and their receptors have been demonstrated to play an importantrolein facilitating immune cell infiltration to the CNS for WNV clearance. The CCR2 chemokine ligands CCL2 and CCL7 are both involved in monocytosis and monocyte accumulation in the brain. However, CCL7 seems to play a larger role in WNV-induced monocytosis and is involved in the efficient recruitment of neutrophils and CD8 ^+^ T cells into the CNS ^29^. CCR5 is required for virologic control, specifically within the CNS cortex. WNV-infected CCR5 ^-/-^ mice had a significant decrease in immune cell infiltrates, increased BBB permeability, and elevated levels of CCR5 ligands ^30^. Several factors are important for promoting chemokine-mediated leukocyte migration. For example, CD22 is essential in the control of WNV infection. It was expressed on a subset of splenic DCIR2 ^+^ DCs in mice, which rapidly expanded early after WNV infection, produced CCL3, and promoted CD8 ^+^ T cell migration into the CNS ^31^. Another study reported that receptor-interacting protein kinase 3 (RIPK3) promotes the production of the chemokines CCL2 and CXCL10 in neurons following WNV infection in mice and this helps to recruit T lymphocytes and inflammatory myeloid cells to the CNS for viral clearance ^32^. Finally, studies in horses infected with a newly emerging WNV strain, WNV _NSW2011_, suggest that early IFN and inflammatory cytokine responses in circulating leukocytes and lymphoid organs are associated with subclinical WNV infection ^33^.

PRR-mediated signaling pathways are also involved in regulating the effector activities of innate immune cells. γδ T cells are important for the early control of WNV dissemination and regulation of adaptive immunity against WNV infection. Following WNV infection, TLR7 provides co-stimulatory signals during TCR activation of γδ T cells ^34^. Furthermore, the dysregulated TLR7 signaling pathways due to aging lead to impaired γδ T cell expansion in old mice vaccinated with an attenuated WNV mutant strain ^35^. The RLR and IFN signaling pathways contribute to the regulation of natural killer cell effector activities and the control of pathological inflammation induced in myeloid cells, respectively ^36^. MAVS expression on hematopoietic cells is critical for regulating the inflammatory response and protecting the host from lethal WNV infection ^37^.

### Adaptive immunity

Mature B cells and WNV-specific antibodies are critical in the control of WNV infection and dissemination. However, a recent study suggests that immature B cells present in B-cell-activating factor receptor-deficient mice also contribute to antiviral immunity and protect the host in passive and active immunizations ^38^. T cells provide long-lasting protection against WNV. Graham *et al*. demonstrated in WNV-infected mice that the regulatory T cell (Treg)-dependent production of transforming growth factor beta is required for the induction of CD103 expression on CD8 ^+^ T cells, thereby generating and maintaining a large pool of WNV-specific resident memory CD8 ^+^ T cells ^39^. Aging is a known risk factor for WNV-induced encephalitis in mice and humans. Old mice display an enhanced vulnerability to WNV infection, partially due to cell-trafficking defects in the draining lymph nodes, which result in delayed T cell recruitment and antigen recognition and an impaired IgM and IgG response ^40^.

PRR-mediated innate immunity plays an important role in regulating T- and B-cell responses during WNV infection. For example, both TLR3- and MyD88-dependent signaling pathways contribute to the development of WNV-specific antibody and B-cell memory responses following immunization with a single-cycle WNV vaccine ^41^. MAVS and TLR7 are both required for T-cell priming but are dispensable for recall T-cell responses following an attenuated WNV strain infection. Instead, the TLR7-independent MyD88 signaling pathways, such as IL-1 receptor (IL-1R) signaling, are involved in memory T-cell development ^42, 43^. Both IL-1R and RLR-mediated innate signaling pathways are required for optimal CD4 ^+^ and CD8 ^+^ T-cell activation and subsequent clearance of WNV in the CNS ^44– 46^. Other innate factors, such as IL-17A, are required for promoting CD8 ^+^ T-cell cytotoxicity and WNV clearance ^47^. Lastly, although the intrinsic MAVS signaling is dispensable for Treg proliferation and suppressive capacity, the overproduction of proinflammatory cytokines generated in MAVS-deficient mice contributes to a failure of Treg expansion ^48^.

## WNV pathogenesis

### WNV entry into the CNS and induction of encephalitis

Following natural transmission to the host via mosquito bites, WNV replicates in keratinocytes and the skin residential DCs, Langerhans cells (LCs). Activated LCs then migrate to local draining lymph nodes from the epidermis, after which viremia begins and WNV disseminates to the kidneys, spleen, and other visceral organs ^49^. Although how WNV enters the CNS is not clearly understood, both hematogenous and transneural pathways have been proposed ^50, 51^. It has also been suggested that WNV crosses the BBB in the hematogenous pathway. WNV PAMPs orchestrate endothelial responses to WNV via competing PRRs, including TLR3-mediated innate immune cytokine signals at the BBB. While proinflammatory cytokines such as TNF-α and IL-1β increase the permeability of endothelial barriers, type I and type III IFNs promote and stabilize the BBB ^17, 20^. Another proinflammatory cytokine, osteopontin (OPN), compromises BBB integrity by recruiting WNV-infected polymorphonuclear neutrophil (PMN) infiltration and facilitates WNV entry via a Trojan horse mechanism ^52^. WNV most likely propagates within the CNS transsynaptically by both anterograde and retrograde axonal transport ^53^. WNV-induced CNS diseases are caused partially by bystander damage from the immune response to virus infection in CNS-resident cells and/or infiltrating leukocytes following systemic immune responses. The trafficking of Ly6C ^hi^ monocytes into the brain was pathogenic, as blocking these cells using anti-very late antigen 4 integrin antibody at the time of observation of the first weight loss and leukocyte influx resulted in long-term survival in mice with lethal encephalitis ^54^. In an *ex vivo* spinal cord slice culture (SCSC) model, it was shown that CNS-resident cells had the capacity to initiate a robust innate immune response against WNV infection in the absence of infiltrating inflammatory cells and systemic immune responses ^55^. Furthermore, treatment with minocycline in WNV-infected SCSC induced the expression of genes associated with the anti-inflammatory activation of microglia while inhibiting the expression of genes associated with proinflammatory microglia activation, and this was protective for multiple CNS cell types ^56^.

Many WNV virulent strains in lineages I and II have been reported to be associated with neurological diseases. Koutango virus (WNVKOU) belongs to lineages outside lineages I and II and was shown to be more virulent in mice than WNVNY99, a known virulent lineage I virus. The enhanced virulence of WNVKOU was associated with its poor viral clearance and the induction of a poor neutralizing antibody response ^57^.

### WNV-induced neurological sequelae and chronic kidney disease

Some WNV convalescent patients develop persistent neurological sequelae and/or chronic kidney disease ^4, 5^. The underlying mechanisms are not clearly understood. Vasek *et al*. recently developed a novel mouse model of human WNV neuroinvasive disease by using a WNV isolate with a point mutation in the NS5 protein (WNV-NS5-E218A), which induces infection with similar survival rates and cognitive dysfunction compared to humans. They demonstrated microglial engulfment of hippocampal CA3 presynaptic terminals via complement during acute WNV infection and after recovery ^58^. Furthermore, in the same mouse model, Garble *et al*. subsequently found that mice that had recovered from West Nile neuroinvasive disease exhibited fewer neuroblasts and increased astrogenesis without recovery of hippocampal neurogenesis. Preferential generation of IL-1 in astrocytes impaired the homeostasis of neuronal progenitor cells ^59^. These results not only suggest a potential mechanism underlying neurocognitive impairment in patients recovering from WNV neuroinvasive disease but also provide potential therapeutic targets.

Increasing evidence suggests that persistent WNV infection also contributes to long-term morbidity. WNV antigen, RNA, or virus particles have been detected in the brain and/or urine of WNV patients ranging from a few months to several years after the initial acute illness ^60, 61^. Small animal models have been developed to study persistent WNV infection. We and others have shown that the inbred C57BL6 mice infected with either the wild-type WNV strain or an isolate cultured from the urine of a persistently infected hamster share some similarity and discrepancy in symptoms and tissue tropism compared to the clinical findings in some WNV convalescent patients with long-term morbidity, including chronic kidney diseases and long-term neurological sequelae ^62, 63^. More recently, the collaborative cross, a population of recombinant inbred mouse strains with high levels of fixed genetic variation, were used to investigate WNV persistence in the brain. Results from this model suggest that the Treg response sufficiently restrains the immune response and leads to WNV persistence in the CNS ^64^.

## Conclusions

Studies in human cell culture and animal models suggest that both innate and adaptive immune responses are important for protecting the host from WNV infection. PRR-mediated innate immune responses are critical for the control of WNV dissemination and viral clearance in the CNS, modulation of BBB integrity, and regulation of the effector functions of innate and adaptive immune cells. Adaptive immunity provides long-lasting protection against WNV. Newly developed animal models have provided important insights into the mechanism underlying neurocognitive impairment in patients recovering from WNV neuroinvasive disease and WNV persistence in the CNS.
